# Editorial: Kinase inhibitors in cancer therapy

**DOI:** 10.3389/fcell.2022.1020297

**Published:** 2022-10-31

**Authors:** Dharmendra Kumar Yadav

**Affiliations:** College of Pharmacy, Gachon University of Medicine and Science, Incheon, South Korea

**Keywords:** Bax/Bcl-2, kinase inhibitors, EGFR pathway, RAS-RAF-MEK-ERK pathway, RET = rearranged during transfection

Protein kinases regulate nearly every aspect of cell life, and changes in their expression or gene mutations cause cancer and other diseases. As many human diseases result from mutations and overexpression of kinases, this enzyme class symbolizes an important targeted strategy for drug development. Kinases are also essential in signaling pathways that regulate tumor cell functions. Kinase dysregulation causes a number of pathophysiological changes that promote cancer cell proliferation and metastasis. This targeted, structure-based drug design is gaining traction; several anticancer drugs targeting specific kinases are in various stages of clinical trials. When compared to a decade ago, the new opportunities, developments, and results in this field are almost unbelievable.

The development of superior drugs that target specific signaling molecules has had a significant impact on the pipelines of pharmaceutical companies, leading to the development of multiple marketed kinase inhibitors. The development of selective small molecules against kinases has the potential to provide greater potency and selectivity than was previously possible.

The authors included in this Research Topic discuss how various classes of kinases are used as potential drug targets, with a particular emphasis on anticancer therapy. The content covers both the challenges and opportunities for future kinase inhibitor development for anticancer therapy. Contributions also discuss how the problem of drug resistance to kinase inhibitors is being addressed, as well as the future of kinase drug discovery. Kinases are currently being studied extensively for the treatment of a variety of life-threatening diseases. This Research Topic brings together several reviews and research articles, contributed by scientists and researchers in the fields of medicinal chemistry and molecular biology, as well as new emerging multi-drug targets.

This Research Topic starts with a research article on “*Hepatic Rupture as the Initial Presentation of an EGFR-Mutated Lung Adenocarcinoma: A Case Report*” by Mirallas et al., which describes a female patient who presented with a metastatic hepatic rupture and was later diagnosed with EGFR-mutated lung adenocarcinoma. Hepatic rupture is a rare complication in patients with solid tumor malignancies, particularly lung adenocarcinomas, and it is associated with a very poor prognosis.

The next article in this Research Topic is a review by Alam et al. examining “*Bax/Bcl-2’s Cascade Is Regulated by the EGFR Pathway: Therapeutic Targeting of Non-Small Cell Lung Cancer*.” The review summarizes recent information on the molecular mechanisms of the Bax/Bcl-2 cascade and the EGFR pathway in NSCLC and targets them for therapeutic implications. This study looked at the therapeutic potential of Bax/Bcl-2/EGFR SMIs, particularly those with higher potency and selectivity, such as gefitinib, EGCG, ABT-737, thymoquinone, quercetin, and venetoclax. Furthermore, they also discuss the EGFR pathway and ongoing *in vitro*, *in vivo*, and clinical studies in NSCLC. Exploration of such inhibitors facilitates future NSCLC treatment and management.

Research by Wu et al. in their contribution, “*Lestaurtinib Has the Potential to Inhibit the Proliferation of Hepatocellular Carcinoma Uncovered by Bioinformatics Analysis and Pharmacological Experiments*,” identifies a new candidate STAT family. This factor is involved in the development of HCC and new treatment Candidate STAT family genes for HCC were found using bioinformatics web resources such as Oncomine, Gene Expression Profiling Interactive Analysis (GEPIA), The Human Protein Atlas (HPA), Tumor Immune Estimation Resource (TIMER), and GSCALite. In this study, the author performed *in vitro* experiments using lestaurtinib as a treatment for patients with liver cancer and verified that lestaurtinib, a tyrosine kinase inhibitor, can inhibit the growth of liver cancer cells. The author explores the effects of lestaurtinib in hepatocellular carcinoma through *in vitro* and *in vivo* systemic studies to provide a new strategy for the treatment of hepatocellular carcinoma. *In vivo* studies focus on addressing how to limit toxicity at optimal biological doses.

The fourth article in this Research Topic, “*Therapeutic Implications of Caffeic Acid in Cancer and Neurological Diseases*,” is a review by Alam et al. that focuses on the chemical, physical, and pharmacological properties of caffeine, including its antioxidant, anti-inflammatory, anticancer, and neuroprotective effects. The features, therapeutic potential, and future possibilities of CA are also discussed by the author.

The fifth article in this Research Topic by Chen et al. identified a novel LDLR-ROS1 fusion in patients with resectable stage IIIA NSCLC, entitled “*Case Report: Adjuvant Crizotinib Therapy Exerted Favorable Survival Benefit in a Resectable Stage IIIA NSCLC Patient With Novel LDLR-ROS1 Fusion*.” After receiving crizotinib as adjuvant therapy, the patient experienced no substantial harmful side effects and maintained recurrence-free survival (RFS) for 29 months. This example supports the use of adjuvant treatment with the ROS1 inhibitor exhibiting clinical survival advantage in ROS1 fusion-positive resected NSCLC, according to the authors’ report of a novel LDLR-ROS1 fusion responding to crizotinib in a patient with lung adenocarcinoma.

The sixth article in this Research Topic is a review by Avery et al. on “*Onco-immunomodulatory properties of pharmacological interference with RAS-RAF-MEK-ERK pathway hyperactivation.*” This article outlines the rationale and concepts for utilizing the immunomodulatory properties of RAS-RAF-MEK-ERK inhibition while emphasizing the role of MEK inhibition in combinatorial and intermittent anemia treatment. The considerable scientific efforts made to address the difficulties encountered during the clinical transition of different therapeutic drugs were also highlighted in the hunt for the most efficient and secure patient- and tumor-tailored treatment strategy.

The seventh article in this Research Topic, “*Case Report: A Lung Adenocarcinoma With Brain Metastasis Harbored Novel MET 14 Skipping Alteration Sensitive to Savolitinib*,” was written by Li et al. and describes a 61-year-old woman who had lung adenocarcinoma and was treated with Savolitinib after having a novel MET exon 14 skipping (c.3004). These two MET changes were also validated by the CytoTest MET/CCP7 FISH Probe (c-MET/CCP7 Ratio:1.41 and mean gene copy number:6) and qPCR, which used the ABI 7500. Savolitinib treatment lasted for 2 months, and the clinical assessment showed a partial response (PR). In conclusion, this discovery not only broadened the range of the MET exon14 variation (METex14). Targeted NGS analysis may enhance the ability to detect MET changes in everyday practice.

The final article in this Research Topic, “*RET Signaling Pathway and RET Inhibitors in Human Cancer*,” by Regua et al. reviews biological understanding of RET signaling, the effects of RET hyperactivity on tumor progression in a variety of tumor types, and the efficacy of RET inhibitors in preclinical and clinical settings.

I would like to thank all authors who contributed to this Research Topic on “*Kinase Inhibitors in Cancer Therapy*.”

